# Outcomes Comparisons Based on Gross National Income: Problems Without Borders?∗

**DOI:** 10.1016/j.jacadv.2023.100397

**Published:** 2023-06-30

**Authors:** David G. Nykanen

**Affiliations:** Orlando Health Arnold Palmer Children’s Hospital, Orlando, Florida, USA

**Keywords:** pediatric cardiac catheterization outcomes

**Figure:**
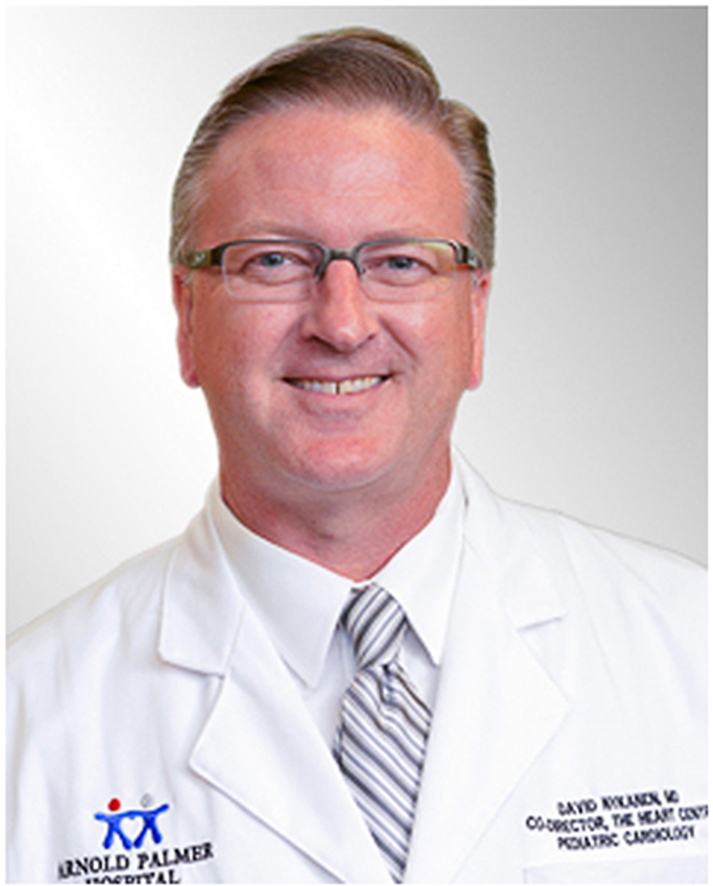

Patients with congenital heart disease represent considerable variation in anatomic detail, physiology, and demographic characteristic that render precatheterization risk assessment and outcomes analysis challenging. Initial efforts in outcomes analysis for this population focused on surgical procedures and specifically addressed only mortality. Furthermore, low volumes of like patients with comparable procedures challenge outcomes analysis. Technological advances over the past 3 decades have resulted in the development of invasive therapeutic transcatheter procedures but outcomes and risk-based analysis has trailed the surgical literature. More recently there has been an effort represented predominantly by academic programs in the United States to address these shortcomings in registry-based research currently represented in large part by the Congenital Cardiac Catheterization Outcomes Project (C3PO), IMproving Pediatric and Adult Congenital Treatments Registry and the Congenital Cardiovascular Interventional Study Consortium.^1^, ^2^, ^3^

In this issue of *JACC: Advances*, Ali et al^4^ report furthering this effort beyond North America to low/middle income countries (LMIC) as defined by gross national income. By utilizing the methodology largely developed by C3PO, the International Quality Improvement Collaborative Congenital Heart Disease Catheterization Registry (IQIC-CHDCR) was “harmonized” to the C3PO registry to allow for comparative analysis including the application of Catheterization for Congenital Heart Disease Adjustment for Risk Method II risk adjustment and Procedural Risk in Congenital Cardiac Catheterization risk stratification^5^^,^^6^ to draw comparison with a high-income country (HIC); represented by data from the United States.

The study is represented by 15 centers in 12 countries with audited data from 3,287 catheterizations submitted to the IQIC-CHDCR over the 24-month period from January 2019 to December 2020. Data collection was reasonably robust, excepting intraprocedural hemodynamics, and there was independent review of adverse events and severity scoring. Outcome measures consisted of mortality during or within 72 hours of the procedure, significant adverse events and procedural efficacy which was a composite of technical success and safety for 5 common interventional procedures (occlusion of atrial septal defect and patent ductus arteriosus, aortic, and pulmonary valvuloplasty and aortic coarctation angioplasty). The majority (93%) of the procedures were elective and 60% included a transcatheter intervention. Case mixture was assigned as minimal risk in 75% of the procedures. Notably 75% of procedures included the use of general anesthesia with 70% of the patients intubated or having a tracheostomy, suggesting advanced airway management and expertise was largely available.

In this cohort 34 patients (1%) died which is notably higher than that experienced in North America. The authors speculate that this may be due to a failure to rescue patients related to immediate access to surgery and advanced mechanical circulatory support. At least 1 center included catheterization on patients with mechanical support thus suggesting that available rescue resources vary between centers. Using Catheterization for Congenital Heart Disease Adjustment for Risk Method II methodology, the standardized rates for serious adverse events and high serious adverse events was 0.60 and 0.72, respectively. Interestingly despite the use of general anesthesia and advanced airway management 28% of adverse events were deemed related to sedation and airway management. It is important to note that 30% (997/3,287) procedures were represented by transcatheter occlusion of a patent ductus arteriosus or an atrial septal defect with very good composite outcomes based on effectiveness and adverse event rate. This represents a substantial proportion of relatively simple interventions in this cohort compared to centers in the United States. This may reflect a practice pattern and referral bias in the LMIC centers wherein one could speculate that preference may favor investment in procedures that offer long term cure rather than ongoing palliation. The skew of the type of procedure represented in the study is in part compensated by risk adjustment but closer examination of the entire cohort is necessary to address treatment biases. When resources are limited the relative cost of implantable devices, disposables, labor and post procedural care have a strong influence on whether one undertakes a surgical or transcatheter approach.

The study purports to compare procedures in LMIC compared to those undertaken in a HIC, namely the United States. However, as the authors appropriately acknowledge, what may have a greater influence is the resources available to individual centers. Participation in the IQIC-CHDCR registry is voluntary and has no direct cost for participation. The demand of local resource associated for data entry and audit as well as motivation to participate may favor high resource programs in LMIC. This is important as the authors concede that participation included approximately one fifth of known centers within LMIC even though participation in the registry has no fee. Even in HIC the local resources required for registry participation may affect generalization of the observed results to all centers as most centers must carefully consider the value added for these quality-based analyses. In this study no information is provided as to the relative contribution of each participating center to the data. With a short 2-year duration and acknowledgment that one third of programs began contributing data in the second year, it is difficult to generalize the findings to all centers in LMIC. This gives pause to generalizing findings by global definitions of country wealth. The ability to participate in multicentered study does vary between centers globally. The availability of preprocedural risk assessment tools such as CRISP (Catheterization RISk score for Pediatrics)^3^ with free access to simple on-line resources may be a benefit for centers with less resource to examine their own programmatic performance in this complex clinical environment.

This work confirms that risk related strategies developed in one part of the world can be utilized to evaluate outcomes irrespective of geographic location. Central to the success of this effort was the collaboration and planning demonstrated in data collection. The collaboration inherent in aligning the data between the IQIC-CHDCR and C3PO registries facilitated data analysis presumably by utilizing the existing analysis strategy. Here the resources of C3PO were able to be extended to IQIC-CHDCR without having to reinvent another analysis strategy.

Intuitively one would expect that methodology that is blind to its country of origin should be equally applicable in other locations. The best way to assess this hypothesis is to mirror data collection and analysis as demonstrated by this work. It should not be a surprise that outcome is due to available skill, resources, and planning rather than specific location. It is gratifying to confirm that currently available outcome measurements also apply outside the borders of North America. This confirms risk stratification strategies can effectively “level the field” when it comes to evaluating outcomes. That is not to say that all environments are equal. Infection, malnourishment, poverty, and individual wealth vary between and within populations both inside and outside of national borders. The authors reference the increased mortality for neonates born with congenital heart disease in LMIC relative to HIC,^7^ but etiology is complex and not the least of which may represent access to care rather than the quality of care. The concept of per capita “income” is a broad measure of a country’s wealth and does not consider distribution within that country’s borders. The best care available is of little use to one that does not possess the ability to access it whether one lives in a LMIC or HIC. More sophisticated determinants of risk in the future may consider true measures of poverty, socioeconomic status, immigration, education, access to health care and others which surely have an influence on short- and long-term outcome.

The authors should be congratulated for demonstrating that it is possible to develop a registry for procedures undertaken in LMIC and that robust analysis yields results that can be utilized to assess procedural data with the goal of improving outcomes. Importantly they have demonstrated that this methodology can be applied globally. Now that this investigation has demonstrated proof of concept, future efforts should focus on availability of specific resources. It is important to understand what resources are most important for successful outcomes, recognizing that allocation of these resources and regionalization may differ with a nation’s economic resources. Achieving health equity is a global problem and not exclusively confined to borders. We have more work to do.

## Funding support and author disclosures

The author has reported that he has no relationships relevant to the contents of this paper to disclose.
